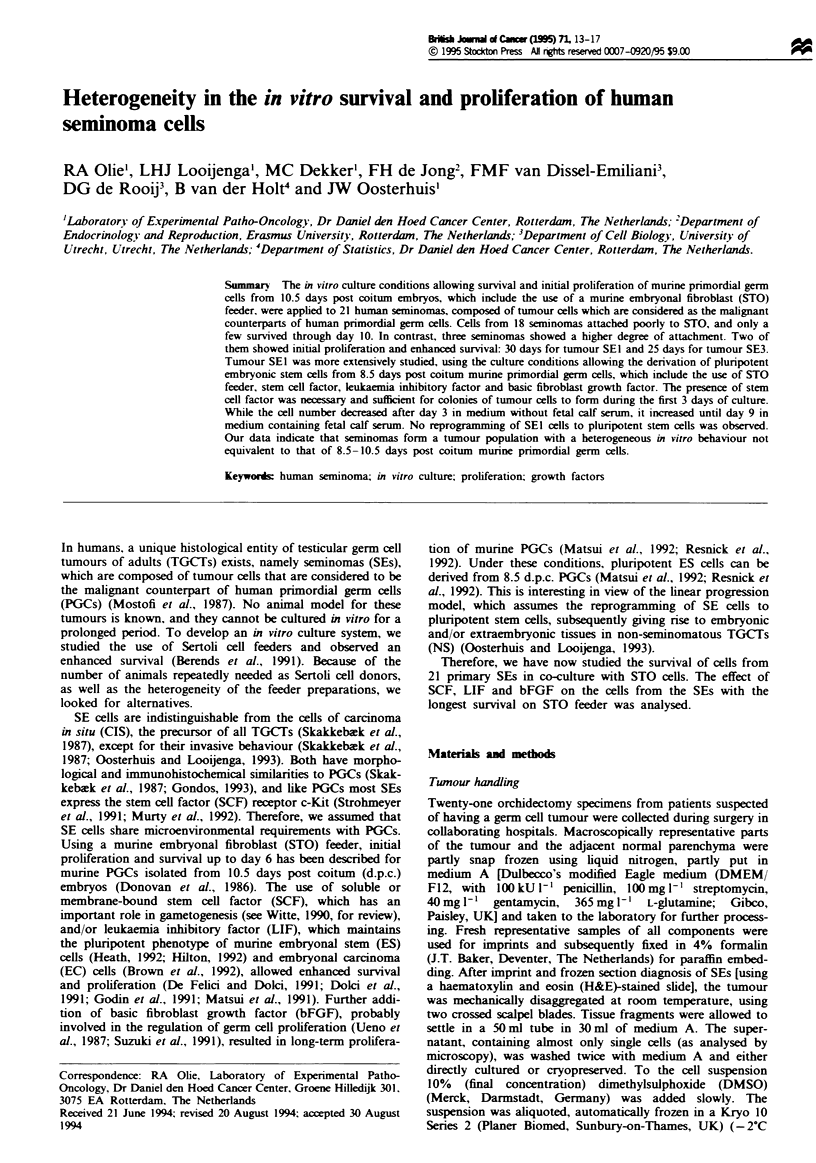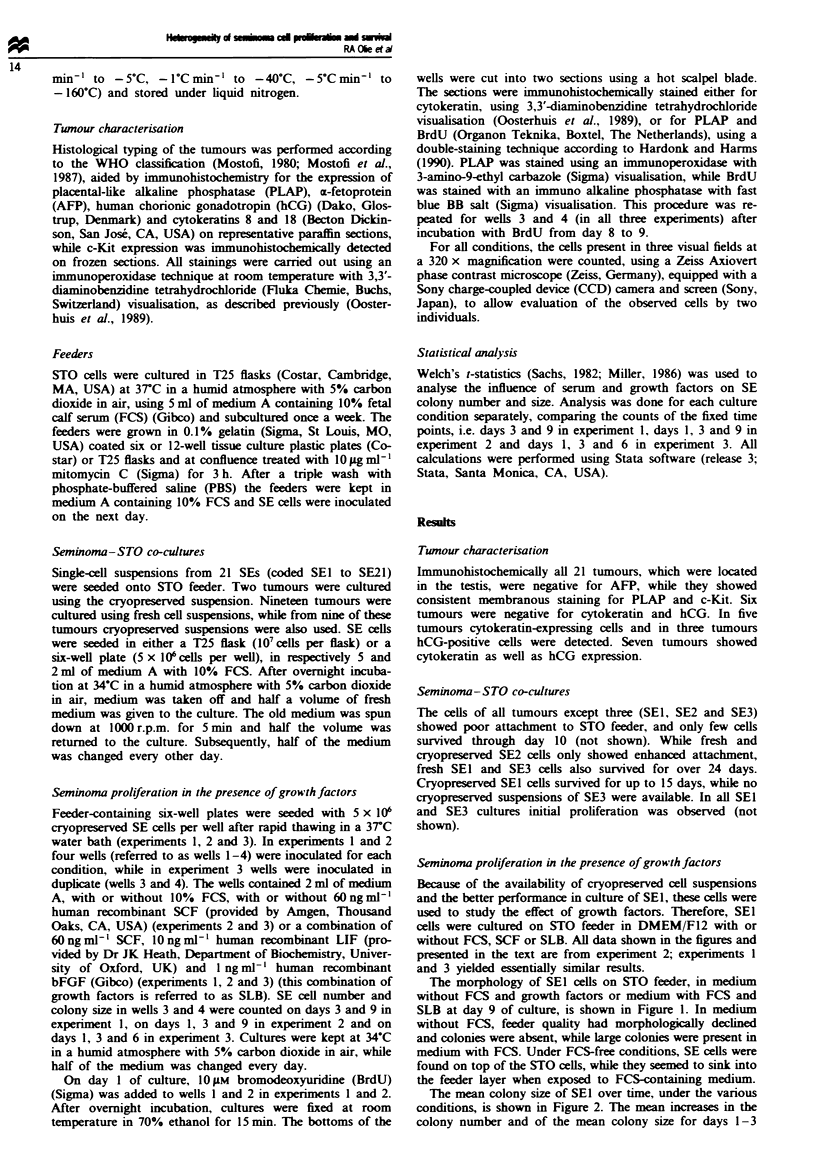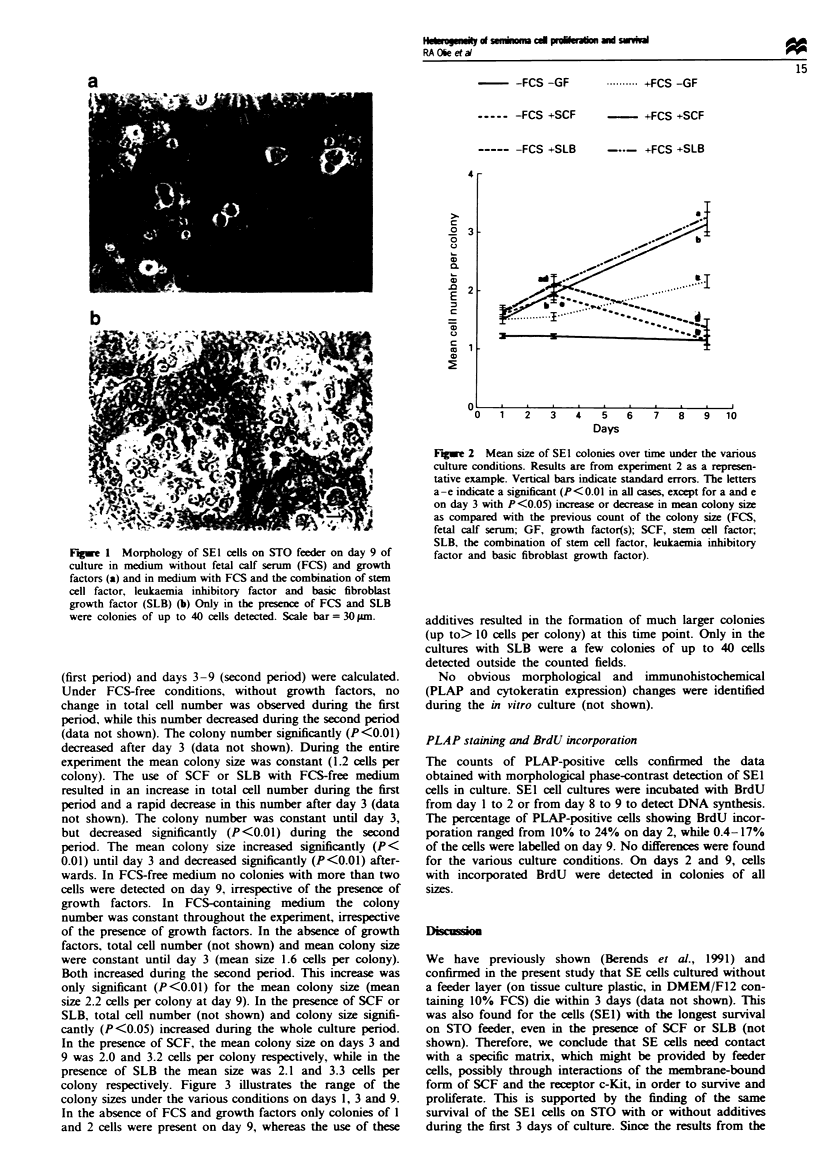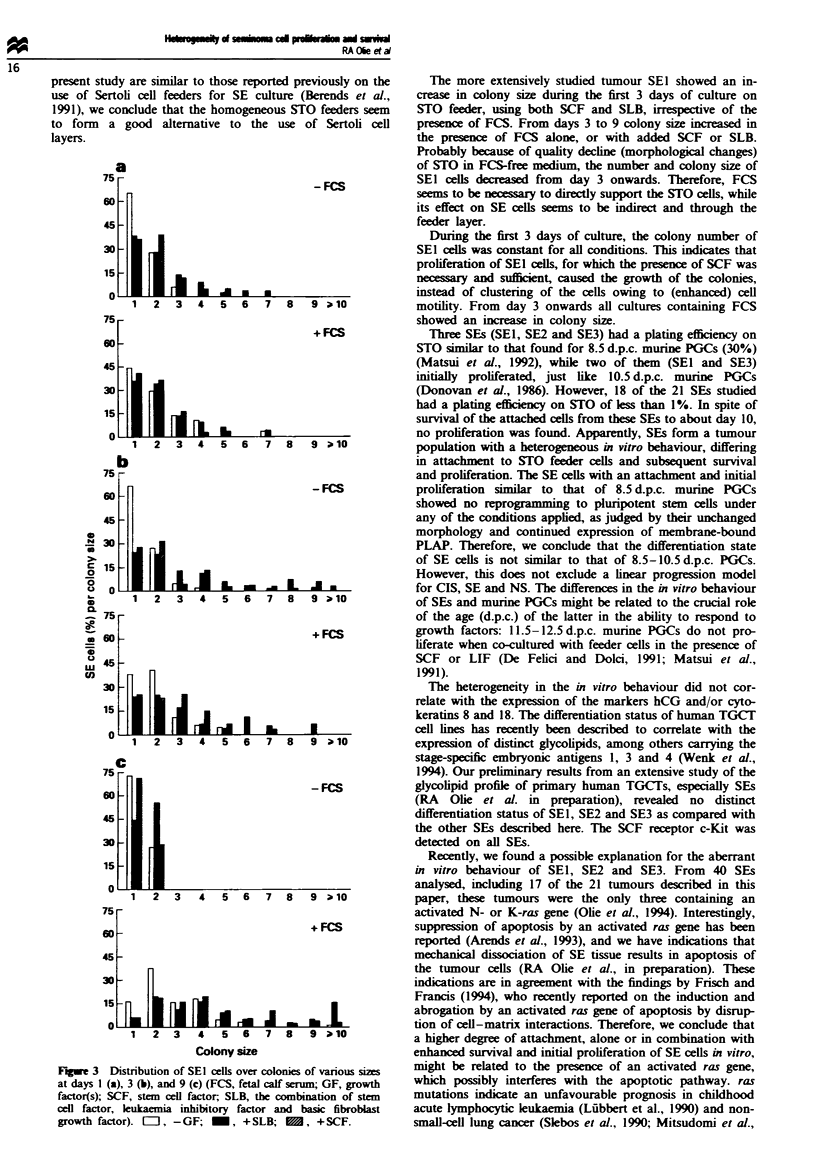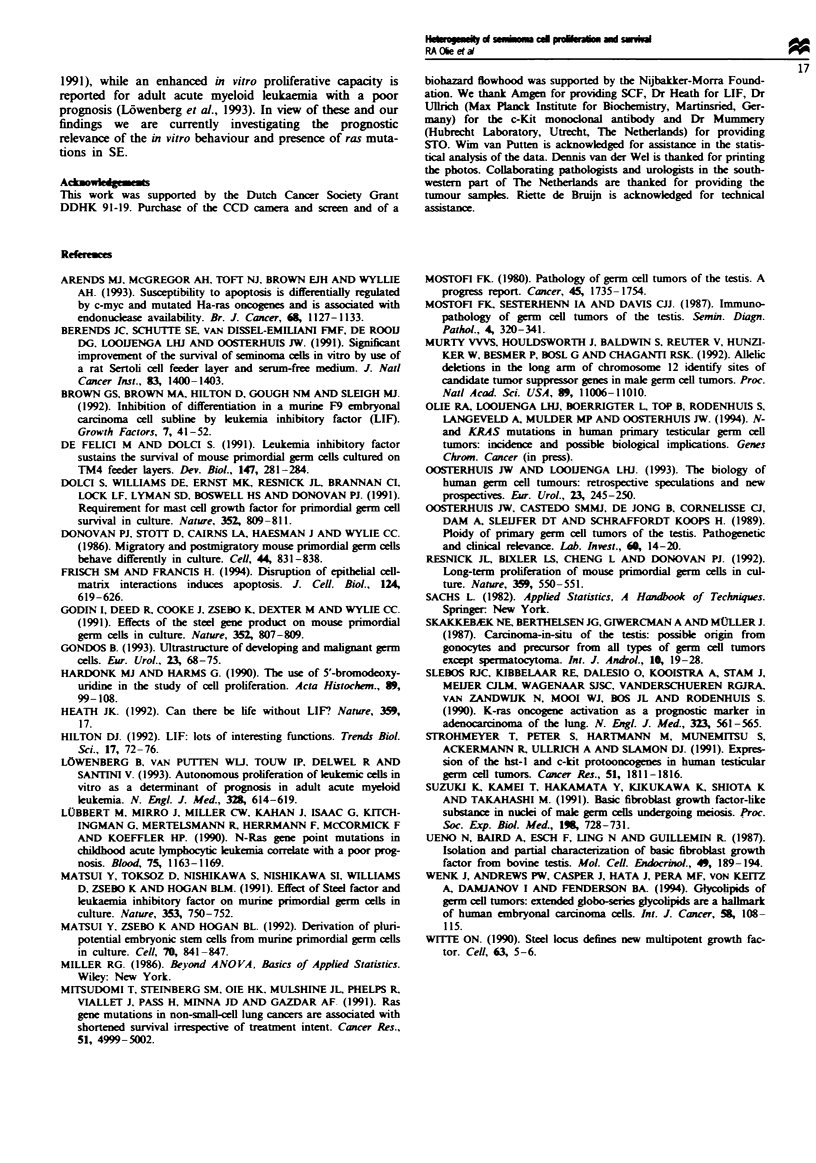# Heterogeneity in the in vitro survival and proliferation of human seminoma cells.

**DOI:** 10.1038/bjc.1995.4

**Published:** 1995-01

**Authors:** R. A. Olie, L. H. Looijenga, M. C. Dekker, F. H. de Jong, F. M. van Dissel-Emiliani, D. G. de Rooij, B. van der Holt, J. W. Oosterhuis

**Affiliations:** Laboratory of Experimental Patho-Oncology, Dr Daniel den Hoed Cancer Center, Rotterdam, The Netherlands.

## Abstract

**Images:**


© 1995 Stockton Press All rights reserved 0007-0920/95 $9.00

### Heterogeneity in the *in vitro* survival and proliferation of human seminoma cells

RA Olie[1], LHJ Looijenga[1], MC Dekker[1], FH de Jong[2], FMF van Dissel-Emiliani[3], DG de Rooij[3], B van der Holt[4] and JW Oosterhuis[1]

[1]*Laboratory of Experimental Patho-Oncology, Dr Daniel den Hoed Cancer Center, Rotterdam, The Netherlands;* [2]*Department of Endocrinology and Reproduction, Erasmus University, Rotterdam, The Netherlands;* [3]*Department of Cell Biology, University of Utrecht, Utrecht, The Netherlands;* [4]*Department of Statistics, Dr Daniel den Hoed Cancer Center, Rotterdam, The Netherlands.*

**Summary** The *in vitro* culture conditions allowing survival and initial proliferation of murine primordial germ cells from 10.5 days post coitum embryos, which include the use of a murine embryonal fibroblast (STO) feeder, were applied to 21 human seminomas, composed of tumour cells which are considered as the malignant counterparts of human primordial germ cells. Cells from 18 seminomas attached poorly to STO, and only a few survived through day 10. In contrast, three seminomas showed a higher degree of attachment. Two of them showed initial proliferation and enhanced survival: 30 days for tumour SE1 and 25 days for tumour SE3. Tumour SE1 was more extensively studied, using the culture conditions allowing the derivation of pluripotent embryonic stem cells from 8.5 days post coitum murine primordial germ cells, which include the use of STO feeder, stem cell factor, leukaemia inhibitory factor and basic fibroblast growth factor. The presence of stem cell factor was necessary and sufficient for colonies of tumour cells to form during the first 3 days of culture. While the cell number decreased after day 3 in medium without fetal calf serum, it increased until day 9 in medium containing fetal calf serum. No reprogramming of SE1 cells to pluripotent stem cells was observed. Our data indicate that seminomas form a tumour population with a heterogeneous *in vitro* behaviour not equivalent to that of 8.5-10.5 days post coitum murine primordial germ cells.

**Keywords:** human seminoma; *in vitro* culture; proliferation; growth factors

In humans, a unique histological entity of testicular germ cell tumours of adults (TGCTs) exists, namely seminomas (SEs), which are composed of tumour cells that are considered to be the malignant counterpart of human primordial germ cells (PGCs) (Mostofi *et al.*, 1987). No animal model for these tumours is known, and they cannot be cultured *in vitro* for a prolonged period. To develop an *in vitro* culture system, we studied the use of Sertoli cell feeders and observed an enhanced survival (Berends *et al.*, 1991). Because of the number of animals repeatedly needed as Sertoli cell donors, as well as the heterogeneity of the feeder preparations, we looked for alternatives.

SE cells are indistinguishable from the cells of carcinoma *in situ* (CIS), the precursor of all TGCTs (Skakkebæk *et al.*, 1987), except for their invasive behaviour (Skakkebæk *et al.*, 1987; Oosterhuis and Looijenga, 1993). Both have morphological and immunohistochemical similarities to PGCs (Skakkebæk *et al.*, 1987; Gondos, 1993), and like PGCs most SEs express the stem cell factor (SCF) receptor c-Kit (Strohmeyer *et al.*, 1991; Murty *et al.*, 1992). Therefore, we assumed that SE cells share microenvironmental requirements with PGCs. Using a murine embryonal fibroblast (STO) feeder, initial proliferation and survival up to day 6 has been described for murine PGCs isolated from 10.5 days post coitum (d.p.c.) embryos (Donovan *et al.*, 1986). The use of soluble or membrane-bound stem cell factor (SCF), which has an important role in gametogenesis (see Witte, 1990, for review), and/or leukaemia inhibitory factor (LIF), which maintains the pluripotent phenotype of murine embryonal stem (ES) cells (Heath, 1992; Hilton, 1992) and embryonal carcinoma (EC) cells (Brown *et al.*, 1992), allowed enhanced survival and proliferation (De Felici and Dolci, 1991; Dolci *et al.*, 1991; Godin *et al.*, 1991; Matsui *et al.*, 1991). Further addition of basic fibroblast growth factor (bFGF), probably involved in the regulation of germ cell proliferation (Ueno *et al.*, 1987; Suzuki *et al.*, 1991), resulted in long-term proliferation of murine PGCs (Matsui *et al.*, 1992; Resnick *et al.*, 1992). Under these conditions, pluripotent ES cells can be derived from 8.5 d.p.c. PGCs (Matsui *et al.*, 1992; Resnick *et al.*, 1992). This is interesting in view of the linear progression model, which assumes the reprogramming of SE cells to pluripotent stem cells, subsequently giving rise to embryonic and/or extraembryonic tissues in non-seminomatous TGCTs (NS) (Oosterhuis and Looijenga, 1993).

Therefore, we have now studied the survival of cells from 21 primary SEs in co-culture with STO cells. The effect of SCF, LIF and bFGF on the cells from the SEs with the longest survival on STO feeder was analysed.

Correspondence: RA Olie, Laboratory of Experimental Patho-Oncology, Dr Daniel den Hoed Cancer Center, Groene Hilledijk 301, 3075 EA Rotterdam, The Netherlands
Received 21 June 1994; revised 20 August 1994; accepted 30 August 1994

### Materials and methods

### *Tumour handling*

Twenty-one orchidectomy specimens from patients suspected of having a germ cell tumour were collected during surgery in collaborating hospitals. Macroscopically representative parts of the tumour and the adjacent normal parenchyma were partly snap frozen using liquid nitrogen, partly put in medium A [Dulbecco's modified Eagle medium (DMEM/F12, with 100 kU $l^{-1}$ penicillin, 100 mg $l^{-1}$ streptomycin, 40 mg $l^{-1}$ gentamycin, 365 mg $l^{-1}$ L-glutamine; Gibco, Paisley, UK] and taken to the laboratory for further processing. Fresh representative samples of all components were used for imprints and subsequently fixed in 4% formalin (J.T. Baker, Deventer, The Netherlands) for paraffin embedding. After imprint and frozen section diagnosis of SEs [using a haematoxylin and eosin (H&E)-stained slide], the tumour was mechanically disaggregated at room temperature, using two crossed scalpel blades. Tissue fragments were allowed to settle in a 50 ml tube in 30 ml of medium A. The supernatant, containing almost only single cells (as analysed by microscopy), was washed twice with medium A and either directly cultured or cryopreserved. To the cell suspension 10% (final concentration) dimethylsulphoxide (DMSO) (Merck, Darmstadt, Germany) was added slowly. The suspension was aliquoted, automatically frozen in a Kryo 10 Series 2 (Planer Biomed, Sunbury-on-Thames, UK) (−2°C

min$^{-1}$ to $-5°C$, $-1°C\,min^{-1}$ to $-40°C$, $-5°C\,min^{-1}$ to $-160°C$) and stored under liquid nitrogen.

*Tumour characterisation*

Histological typing of the tumours was performed according to the WHO classification (Mostofi, 1980; Mostofi *et al.*, 1987), aided by immunohistochemistry for the expression of placental-like alkaline phosphatase (PLAP), α-fetoprotein (AFP), human chorionic gonadotropin (hCG) (Dako, Glostrup, Denmark) and cytokeratins 8 and 18 (Becton Dickinson, San José, CA, USA) on representative paraffin sections, while c-Kit expression was immunohistochemically detected on frozen sections. All stainings were carried out using an immunoperoxidase technique at room temperature with 3,3'-diaminobenzidine tetrahydrochloride (Fluka Chemie, Buchs, Switzerland) visualisation, as described previously (Oosterhuis *et al.*, 1989).

*Feeders*

STO cells were cultured in T25 flasks (Costar, Cambridge, MA, USA) at 37°C in a humid atmosphere with 5% carbon dioxide in air, using 5 ml of medium A containing 10% fetal calf serum (FCS) (Gibco) and subcultured once a week. The feeders were grown in 0.1% gelatin (Sigma, St Louis, MO, USA) coated six or 12-well tissue culture plastic plates (Costar) or T25 flasks and at confluence treated with 10 μg ml$^{-1}$ mitomycin C (Sigma) for 3 h. After a triple wash with phosphate-buffered saline (PBS) the feeders were kept in medium A containing 10% FCS and SE cells were inoculated on the next day.

*Seminoma–STO co-cultures*

Single-cell suspensions from 21 SEs (coded SE1 to SE21) were seeded onto STO feeder. Two tumours were cultured using the cryopreserved suspension. Nineteen tumours were cultured using fresh cell suspensions, while from nine of these tumours cryopreserved suspensions were also used. SE cells were seeded in either a T25 flask ($10^7$ cells per flask) or a six-well plate ($5 \times 10^6$ cells per well), in respectively 5 and 2 ml of medium A with 10% FCS. After overnight incubation at 34°C in a humid atmosphere with 5% carbon dioxide in air, medium was taken off and half a volume of fresh medium was given to the culture. The old medium was spun down at 1000 r.p.m. for 5 min and half the volume was returned to the culture. Subsequently, half of the medium was changed every other day.

*Seminoma proliferation in the presence of growth factors*

Feeder-containing six-well plates were seeded with $5 \times 10^6$ cryopreserved SE cells per well after rapid thawing in a 37°C water bath (experiments 1, 2 and 3). In experiments 1 and 2 four wells (referred to as wells 1–4) were inoculated for each condition, while in experiment 3 wells were inoculated in duplicate (wells 3 and 4). The wells contained 2 ml of medium A, with or without 10% FCS, with or without 60 ng ml$^{-1}$ human recombinant SCF (provided by Amgen, Thousand Oaks, CA, USA) (experiments 2 and 3) or a combination of 60 ng ml$^{-1}$ SCF, 10 ng ml$^{-1}$ human recombinant LIF (provided by Dr JK Heath, Department of Biochemistry, University of Oxford, UK) and 1 ng ml$^{-1}$ human recombinant bFGF (Gibco) (experiments 1, 2 and 3) (this combination of growth factors is referred to as SLB). SE cell number and colony size in wells 3 and 4 were counted on days 3 and 9 in experiment 1, on days 1, 3 and 9 in experiment 2 and on days 1, 3 and 6 in experiment 3. Cultures were kept at 34°C in a humid atmosphere with 5% carbon dioxide in air, while half of the medium was changed every day.

On day 1 of culture, 10 μM bromodeoxyuridine (BrdU) (Sigma) was added to wells 1 and 2 in experiments 1 and 2. After overnight incubation, cultures were fixed at room temperature in 70% ethanol for 15 min. The bottoms of the wells were cut into two sections using a hot scalpel blade. The sections were immunohistochemically stained either for cytokeratin, using 3,3'-diaminobenzidine tetrahydrochloride visualisation (Oosterhuis *et al.*, 1989), or for PLAP and BrdU (Organon Teknika, Boxtel, The Netherlands), using a double-staining technique according to Hardonk and Harms (1990). PLAP was stained using an immunoperoxidase with 3-amino-9-ethyl carbazole (Sigma) visualisation, while BrdU was stained with an immuno alkaline phosphatase with fast blue BB salt (Sigma) visualisation. This procedure was repeated for wells 3 and 4 (in all three experiments) after incubation with BrdU from day 8 to 9.

For all conditions, the cells present in three visual fields at a 320 × magnification were counted, using a Zeiss Axiovert phase contrast microscope (Zeiss, Germany), equipped with a Sony charge-coupled device (CCD) camera and screen (Sony, Japan), to allow evaluation of the observed cells by two individuals.

*Statistical analysis*

Welch's *t*-statistics (Sachs, 1982; Miller, 1986) was used to analyse the influence of serum and growth factors on SE colony number and size. Analysis was done for each culture condition separately, comparing the counts of the fixed time points, i.e. days 3 and 9 in experiment 1, days 1, 3 and 9 in experiment 2 and days 1, 3 and 6 in experiment 3. All calculations were performed using Stata software (release 3; Stata, Santa Monica, CA, USA).

### Results

*Tumour characterisation*

Immunohistochemically all 21 tumours, which were located in the testis, were negative for AFP, while they showed consistent membranous staining for PLAP and c-Kit. Six tumours were negative for cytokeratin and hCG. In five tumours cytokeratin-expressing cells and in three tumours hCG-positive cells were detected. Seven tumours showed cytokeratin as well as hCG expression.

*Seminoma–STO co-cultures*

The cells of all tumours except three (SE1, SE2 and SE3) showed poor attachment to STO feeder, and only few cells survived through day 10 (not shown). While fresh and cryopreserved SE2 cells only showed enhanced attachment, fresh SE1 and SE3 cells also survived for over 24 days. Cryopreserved SE1 cells survived for up to 15 days, while no cryopreserved suspensions of SE3 were available. In all SE1 and SE3 cultures initial proliferation was observed (not shown).

*Seminoma proliferation in the presence of growth factors*

Because of the availability of cryopreserved cell suspensions and the better performance in culture of SE1, these cells were used to study the effect of growth factors. Therefore, SE1 cells were cultured on STO feeder in DMEM/F12 with or without FCS, SCF or SLB. All data shown in the figures and presented in the text are from experiment 2; experiments 1 and 3 yielded essentially similar results.

The morphology of SE1 cells on STO feeder, in medium without FCS and growth factors or medium with FCS and SLB at day 9 of culture, is shown in Figure 1. In medium without FCS, feeder quality had morphologically declined and colonies were absent, while large colonies were present in medium with FCS. Under FCS-free conditions, SE cells were found on top of the STO cells, while they seemed to sink into the feeder layer when exposed to FCS-containing medium.

The mean colony size of SE1 over time, under the various conditions, is shown in Figure 2. The mean increases in the colony number and of the mean colony size for days 1–3

**Figure 1** Morphology of SE1 cells on STO feeder on day 9 of culture in medium without fetal calf serum (FCS) and growth factors (**a**) and in medium with FCS and the combination of stem cell factor, leukaemia inhibitory factor and basic fibroblast growth factor (SLB) (**b**) Only in the presence of FCS and SLB were colonies of up to 40 cells detected. Scale bar = 30 μm.

**Figure 2** Mean size of SE1 colonies over time under the various culture conditions. Results are from experiment 2 as a representative example. Vertical bars indicate standard errors. The letters a–e indicate a significant ($P<0.01$ in all cases, except for a and e on day 3 with $P<0.05$) increase or decrease in mean colony size as compared with the previous count of the colony size (FCS, fetal calf serum; GF, growth factor(s); SCF, stem cell factor; SLB, the combination of stem cell factor, leukaemia inhibitory factor and basic fibroblast growth factor).

(first period) and days 3–9 (second period) were calculated. Under FCS-free conditions, without growth factors, no change in total cell number was observed during the first period, while this number decreased during the second period (data not shown). The colony number significantly ($P<0.01$) decreased after day 3 (data not shown). During the entire experiment the mean colony size was constant (1.2 cells per colony). The use of SCF or SLB with FCS-free medium resulted in an increase in total cell number during the first period and a rapid decrease in this number after day 3 (data not shown). The colony number was constant until day 3, but decreased significantly ($P<0.01$) during the second period. The mean colony size increased significantly ($P<0.01$) until day 3 and decreased significantly ($P<0.01$) afterwards. In FCS-free medium no colonies with more than two cells were detected on day 9, irrespective of the presence of growth factors. In FCS-containing medium the colony number was constant throughout the experiment, irrespective of the presence of growth factors. In the absence of growth factors, total cell number (not shown) and mean colony size were constant until day 3 (mean size 1.6 cells per colony). Both increased during the second period. This increase was only significant ($P<0.01$) for the mean colony size (mean size 2.2 cells per colony at day 9). In the presence of SCF or SLB, total cell number (not shown) and colony size significantly ($P<0.05$) increased during the whole culture period. In the presence of SCF, the mean colony size on days 3 and 9 was 2.0 and 3.2 cells per colony respectively, while in the presence of SLB the mean size was 2.1 and 3.3 cells per colony respectively. Figure 3 illustrates the range of the colony sizes under the various conditions on days 1, 3 and 9. In the absence of FCS and growth factors only colonies of 1 and 2 cells were present on day 9, whereas the use of these additives resulted in the formation of much larger colonies (up to > 10 cells per colony) at this time point. Only in the cultures with SLB were a few colonies of up to 40 cells detected outside the counted fields.

No obvious morphological and immunohistochemical (PLAP and cytokeratin expression) changes were identified during the *in vitro* culture (not shown).

### *PLAP staining and BrdU incorporation*

The counts of PLAP-positive cells confirmed the data obtained with morphological phase-contrast detection of SE1 cells in culture. SE1 cell cultures were incubated with BrdU from day 1 to 2 or from day 8 to 9 to detect DNA synthesis. The percentage of PLAP-positive cells showing BrdU incorporation ranged from 10% to 24% on day 2, while 0.4–17% of the cells were labelled on day 9. No differences were found for the various culture conditions. On days 2 and 9, cells with incorporated BrdU were detected in colonies of all sizes.

### Discussion

We have previously shown (Berends *et al.*, 1991) and confirmed in the present study that SE cells cultured without a feeder layer (on tissue culture plastic, in DMEM/F12 containing 10% FCS) die within 3 days (data not shown). This was also found for the cells (SE1) with the longest survival on STO feeder, even in the presence of SCF or SLB (not shown). Therefore, we conclude that SE cells need contact with a specific matrix, which might be provided by feeder cells, possibly through interactions of the membrane-bound form of SCF and the receptor c-Kit, in order to survive and proliferate. This is supported by the finding of the same survival of the SE1 cells on STO with or without additives during the first 3 days of culture. Since the results from the present study are similar to those reported previously on the use of Sertoli cell feeders for SE culture (Berends *et al.*, 1991), we conclude that the homogeneous STO feeders seem to form a good alternative to the use of Sertoli cell layers.

**Figure 3** Distribution of SE1 cells over colonies of various sizes at days 1 (**a**), 3 (**b**), and 9 (**c**) (FCS, fetal calf serum; GF, growth factor(s); SCF, stem cell factor; SLB, the combination of stem cell factor, leukaemia inhibitory factor and basic fibroblast growth factor). □, –GF; ■, +SLB; ▨, +SCF.

The more extensively studied tumour SE1 showed an increase in colony size during the first 3 days of culture on STO feeder, using both SCF and SLB, irrespective of the presence of FCS. From days 3 to 9 colony size increased in the presence of FCS alone, or with added SCF or SLB. Probably because of quality decline (morphological changes) of STO in FCS-free medium, the number and colony size of SE1 cells decreased from day 3 onwards. Therefore, FCS seems to be necessary to directly support the STO cells, while its effect on SE cells seems to be indirect and through the feeder layer.

During the first 3 days of culture, the colony number of SE1 cells was constant for all conditions. This indicates that proliferation of SE1 cells, for which the presence of SCF was necessary and sufficient, caused the growth of the colonies, instead of clustering of the cells owing to (enhanced) cell motility. From day 3 onwards all cultures containing FCS showed an increase in colony size.

Three SEs (SE1, SE2 and SE3) had a plating efficiency on STO similar to that found for 8.5 d.p.c. murine PGCs (30%) (Matsui *et al.*, 1992), while two of them (SE1 and SE3) initially proliferated, just like 10.5 d.p.c. murine PGCs (Donovan *et al.*, 1986). However, 18 of the 21 SEs studied had a plating efficiency on STO of less than 1%. In spite of survival of the attached cells from these SEs to about day 10, no proliferation was found. Apparently, SEs form a tumour population with a heterogeneous *in vitro* behaviour, differing in attachment to STO feeder cells and subsequent survival and proliferation. The SE cells with an attachment and initial proliferation similar to that of 8.5 d.p.c. murine PGCs showed no reprogramming to pluripotent stem cells under any of the conditions applied, as judged by their unchanged morphology and continued expression of membrane-bound PLAP. Therefore, we conclude that the differentiation state of SE cells is not similar to that of 8.5–10.5 d.p.c. PGCs. However, this does not exclude a linear progression model for CIS, SE and NS. The differences in the *in vitro* behaviour of SEs and murine PGCs might be related to the crucial role of the age (d.p.c.) of the latter in the ability to respond to growth factors: 11.5–12.5 d.p.c. murine PGCs do not proliferate when co-cultured with feeder cells in the presence of SCF or LIF (De Felici and Dolci, 1991; Matsui *et al.*, 1991).

The heterogeneity in the *in vitro* behaviour did not correlate with the expression of the markers hCG and/or cytokeratins 8 and 18. The differentiation status of human TGCT cell lines has recently been described to correlate with the expression of distinct glycolipids, among others carrying the stage-specific embryonic antigens 1, 3 and 4 (Wenk *et al.*, 1994). Our preliminary results from an extensive study of the glycolipid profile of primary human TGCTs, especially SEs (RA Olie *et al.* in preparation), revealed no distinct differentiation status of SE1, SE2 and SE3 as compared with the other SEs described here. The SCF receptor c-Kit was detected on all SEs.

Recently, we found a possible explanation for the aberrant *in vitro* behaviour of SE1, SE2 and SE3. From 40 SEs analysed, including 17 of the 21 tumours described in this paper, these tumours were the only three containing an activated N- or K-*ras* gene (Olie *et al.*, 1994). Interestingly, suppression of apoptosis by an activated *ras* gene has been reported (Arends *et al.*, 1993), and we have indications that mechanical dissociation of SE tissue results in apoptosis of the tumour cells (RA Olie *et al.*, in preparation). These indications are in agreement with the findings by Frisch and Francis (1994), who recently reported on the induction and abrogation by an activated *ras* gene of apoptosis by disruption of cell–matrix interactions. Therefore, we conclude that a higher degree of attachment, alone or in combination with enhanced survival and initial proliferation of SE cells *in vitro*, might be related to the presence of an activated *ras* gene, which possibly interferes with the apoptotic pathway. *ras* mutations indicate an unfavourable prognosis in childhood acute lymphocytic leukaemia (Lübbert et al., 1990) and non-small-cell lung cancer (Slebos *et al.*, 1990; Mitsudomi *et al.*,

1991), while an enhanced *in vitro* proliferative capacity is reported for adult acute myeloid leukaemia with a poor prognosis (Löwenberg *et al.*, 1993). In view of these and our findings we are currently investigating the prognostic relevance of the *in vitro* behaviour and presence of *ras* mutations in SE.

**Acknowledgements**
This work was supported by the Dutch Cancer Society Grant DDHK 91-19. Purchase of the CCD camera and screen and of a biohazard flowhood was supported by the Nijbakker-Morra Foundation. We thank Amgen for providing SCF, Dr Heath for LIF, Dr Ullrich (Max Planck Institute for Biochemistry, Martinsried, Germany) for the c-Kit monoclonal antibody and Dr Mummery (Hubrecht Laboratory, Utrecht, The Netherlands) for providing STO. Wim van Putten is acknowledged for assistance in the statistical analysis of the data. Dennis van der Wel is thanked for printing the photos. Collaborating pathologists and urologists in the southwestern part of The Netherlands are thanked for providing the tumour samples. Riette de Bruijn is acknowledged for technical assistance.

**References**

ARENDS MJ, MCGREGOR AH, TOFT NJ, BROWN EJH AND WYLLIE AH. (1993). Susceptibility to apoptosis is differentially regulated by c-myc and mutated Ha-ras oncogenes and is associated with endonuclease availability. *Br. J. Cancer*, **68**, 1127–1133.

BERENDS JC, SCHUTTE SE, VAN DISSEL-EMILIANI FMF, DE ROOIJ DG, LOOIJENGA LHJ AND OOSTERHUIS JW. (1991). Significant improvement of the survival of seminoma cells in vitro by use of a rat Sertoli cell feeder layer and serum-free medium. *J. Natl Cancer Inst.*, **83**, 1400–1403.

BROWN GS, BROWN MA, HILTON D, GOUGH NM AND SLEIGH MJ. (1992). Inhibition of differentiation in a murine F9 embryonal carcinoma cell subline by leukemia inhibitory factor (LIF). *Growth Factors*, **7**, 41–52.

DE FELICI M AND DOLCI S. (1991). Leukemia inhibitory factor sustains the survival of mouse primordial germ cells cultured on TM4 feeder layers. *Dev. Biol.*, **147**, 281–284.

DOLCI S, WILLIAMS DE, ERNST MK, RESNICK JL, BRANNAN CI, LOCK LF, LYMAN SD, BOSWELL HS AND DONOVAN PJ. (1991). Requirement for mast cell growth factor for primordial germ cell survival in culture. *Nature*, **352**, 809–811.

DONOVAN PJ, STOTT D, CAIRNS LA, HAESMAN J AND WYLIE CC. (1986). Migratory and postmigratory mouse primordial germ cells behave differently in culture. *Cell*, **44**, 831–838.

FRISCH SM AND FRANCIS H. (1994). Disruption of epithelial cell-matrix interactions induces apoptosis. *J. Cell. Biol.*, **124**, 619–626.

GODIN I, DEED R, COOKE J, ZSEBO K, DEXTER M AND WYLIE CC. (1991). Effects of the steel gene product on mouse primordial germ cells in culture. *Nature*, **352**, 807–809.

GONDOS B. (1993). Ultrastructure of developing and malignant germ cells. *Eur. Urol.*, **23**, 68–75.

HARDONK MJ AND HARMS G. (1990). The use of 5′-bromodeoxyuridine in the study of cell proliferation. *Acta Histochem.*, **89**, 99–108.

HEATH JK. (1992). Can there be life without LIF? *Nature*, **359**, 17.

HILTON DJ. (1992). LIF: lots of interesting functions. *Trends Biol. Sci.*, **17**, 72–76.

LÖWENBERG B, VAN PUTTEN WLJ, TOUW IP, DELWEL R AND SANTINI V. (1993). Autonomous proliferation of leukemic cells in vitro as a determinant of prognosis in adult acute myeloid leukemia. *N. Engl. J. Med.*, **328**, 614–619.

LÜBBERT M, MIRRO J, MILLER CW, KAHAN J, ISAAC G, KITCHINGMAN G, MERTELSMANN R, HERRMANN F, MCCORMICK F AND KOEFFLER HP. (1990). N-Ras gene point mutations in childhood acute lymphocytic leukemia correlate with a poor prognosis. *Blood*, **75**, 1163–1169.

MATSUI Y, TOKSOZ D, NISHIKAWA S, NISHIKAWA SI, WILLIAMS D, ZSEBO K AND HOGAN BLM. (1991). Effect of Steel factor and leukaemia inhibitory factor on murine primordial germ cells in culture. *Nature*, **353**, 750–752.

MATSUI Y, ZSEBO K AND HOGAN BL. (1992). Derivation of pluripotential embryonic stem cells from murine primordial germ cells in culture. *Cell*, **70**, 841–847.

MILLER RG. (1986). *Beyond ANOVA, Basics of Applied Statistics*. Wiley: New York.

MITSUDOMI T, STEINBERG SM, OIE HK, MULSHINE JL, PHELPS R, VIALLET J, PASS H, MINNA JD AND GAZDAR AF. (1991). Ras gene mutations in non-small-cell lung cancers are associated with shortened survival irrespective of treatment intent. *Cancer Res.*, **51**, 4999–5002.

MOSTOFI FK. (1980). Pathology of germ cell tumors of the testis. A progress report. *Cancer*, **45**, 1735–1754.

MOSTOFI FK, SESTERHENN IA AND DAVIS CJJ. (1987). Immunopathology of germ cell tumors of the testis. *Semin. Diagn. Pathol.*, **4**, 320–341.

MURTY VVVS, HOULDSWORTH J, BALDWIN S, REUTER V, HUNZIKER W, BESMER P, BOSL G AND CHAGANTI RSK. (1992). Allelic deletions in the long arm of chromosome 12 identify sites of candidate tumor suppressor genes in male germ cell tumors. *Proc. Natl Acad. Sci. USA*, **89**, 11006–11010.

OLIE RA, LOOIJENGA LHJ, BOERRIGTER L, TOP B, RODENHUIS S, LANGEVELD A, MULDER MP AND OOSTERHUIS JW. (1994). *N*- and *KRAS* mutations in human primary testicular germ cell tumors: incidence and possible biological implications. *Genes Chrom. Cancer* (in press).

OOSTERHUIS JW AND LOOIJENGA LHJ. (1993). The biology of human germ cell tumours: retrospective speculations and new prospectives. *Eur. Urol.*, **23**, 245–250.

OOSTERHUIS JW, CASTEDO SMMJ, DE JONG B, CORNELISSE CJ, DAM A, SLEIJFER DT AND SCHRAFFORDT KOOPS H. (1989). Ploidy of primary germ cell tumors of the testis. Pathogenetic and clinical relevance. *Lab. Invest.*, **60**, 14–20.

RESNICK JL, BIXLER LS, CHENG L AND DONOVAN PJ. (1992). Long-term proliferation of mouse primordial germ cells in culture. *Nature*, **359**, 550–551.

SACHS L. (1982). *Applied Statistics, A Handbook of Techniques*. Springer: New York.

SKAKKEBÆK NE, BERTHELSEN JG, GIWERCMAN A AND MÜLLER J. (1987). Carcinoma-in-situ of the testis: possible origin from gonocytes and precursor from all types of germ cell tumors except spermatocytoma. *Int. J. Androl.*, **10**, 19–28.

SLEBOS RJC, KIBBELAAR RE, DALESIO O, KOOISTRA A, STAM J, MEIJER CJLM, WAGENAAR SJSC, VANDERSCHUEREN RGJRA, VAN ZANDWIJK N, MOOI WJ, BOS JL AND RODENHUIS S. (1990). K-ras oncogene activation as a prognostic marker in adenocarcinoma of the lung. *N. Engl. J. Med.*, **323**, 561–565.

STROHMEYER T, PETER S, HARTMANN M, MUNEMITSU S, ACKERMANN R, ULLRICH A AND SLAMON DJ. (1991). Expression of the hst-1 and c-kit protooncogenes in human testicular germ cell tumors. *Cancer Res.*, **51**, 1811–1816.

SUZUKI K, KAMEI T, HAKAMATA Y, KIKUKAWA K, SHIOTA K AND TAKAHASHI M. (1991). Basic fibroblast growth factor-like substance in nuclei of male germ cells undergoing meiosis. *Proc. Soc. Exp. Biol. Med.*, **198**, 728–731.

UENO N, BAIRD A, ESCH F, LING N AND GUILLEMIN R. (1987). Isolation and partial characterization of basic fibroblast growth factor from bovine testis. *Mol. Cell. Endocrinol.*, **49**, 189–194.

WENK J, ANDREWS PW, CASPER J, HATA J, PERA MF, VON KEITZ A, DAMJANOV I AND FENDERSON BA. (1994). Glycolipids of germ cell tumors: extended globo-series glycolipids are a hallmark of human embryonal carcinoma cells. *Int. J. Cancer*, **58**, 108–115.

WITTE ON. (1990). Steel locus defines new multipotent growth factor. *Cell*, **63**, 5–6.